# Root Zone Sensors for Irrigation Management in Intensive Agriculture

**DOI:** 10.3390/s90402809

**Published:** 2009-04-21

**Authors:** Alberto Pardossi, Luca Incrocci, Giorgio Incrocci, Fernando Malorgio, Piero Battista, Laura Bacci, Bernardo Rapi, Paolo Marzialetti, Jochen Hemming, Jos Balendonck

**Affiliations:** 1 Dipartimento di Biologia delle Piante Agrarie, University of Pisa, Viale delle Piagge 23, 56124-I Pisa, Italy; E-Mails: incrocci@agr.unipi.it (L.I.); g.incrocci@inwind.it (G.I.); fmalorgio@agr.unipi.it (F.M.); 2 Istituto di Biometeorologia,Via Caproni 8, 50145 Firenze, Italy; E-Mails: p.battista@ibimet.cnr.it (P.B.); l.bacci@ibimet.cnr.it (L.B.); b.rapi@ibimet.cnr.it (B.R.); 3 Centro Sperimentale per il Vivaimo (CESPEVI), Via Ciliegiole 99, 51100 Pistoia, Italy; E-Mail: info@cespevi.it (P.M.); 4 Wageningen University and Research Center, Greenhouse Horticulture, Droevendaalsesteeg 1, 6708 PB Wageningen, the Netherlands; E-Mails: Jochen.Hemming@wur.nl (J.H.); jos.balendonck@wur.nl (J.B.)

**Keywords:** Dielectric soil moisture sensors, irrigation efficiency, irrigation scheduling, smart water application technology, soil matric potential, tensiometer, wireless sensor network

## Abstract

Crop irrigation uses more than 70% of the world’s water, and thus, improving irrigation efficiency is decisive to sustain the food demand from a fast-growing world population. This objective may be accomplished by cultivating more water-efficient crop species and/or through the application of efficient irrigation systems, which includes the implementation of a suitable method for precise scheduling. At the farm level, irrigation is generally scheduled based on the grower’s experience or on the determination of soil water balance (weather-based method). An alternative approach entails the measurement of soil water status. Expensive and sophisticated root zone sensors (RZS), such as neutron probes, are available for the use of soil and plant scientists, while cheap and practical devices are needed for irrigation management in commercial crops. The paper illustrates the main features of RZS’ (for both soil moisture and salinity) marketed for the irrigation industry and discusses how such sensors may be integrated in a wireless network for computer-controlled irrigation and used for innovative irrigation strategies, such as deficit or dual-water irrigation. The paper also consider the main results of recent or current research works conducted by the authors in Tuscany (Italy) on the irrigation management of container-grown ornamental plants, which is an important agricultural sector in Italy.

## 1. Introduction

According to FAO [1], nearly 40% of the world’s food is produced by irrigated agriculture, which covers about 250 million hectares (corresponding to 17% of the total arable land) and is the major user of fresh water, accounting for 70% (on average and up to 90% in many countries) of worldwide water withdrawn for human use. In Italy, irrigation accounts for about 50% of total water use and irrigated agriculture covers about 27% of usable farmland [2].

Consequently, improving irrigation water use efficiency (i.e., the ratio between applied water and crop yield) is decisive to satisfy the increased world demand for food and other agricultural products. This objective may be accomplished by cultivating more water-efficient crops (as developed by means of conventional or recombinant DNA-based breeding) and/or through the application of efficient irrigation technology. Despite the efforts made in recent years, irrigation efficiency (i.e., the amount of water stored in the crop root zone compared to the amount of applied water) is still unsatisfactory (less than 40%) [1] and its improvement is a key goal for the future.

Irrigation efficiency depends on the type of irrigation used (for instance, surface irrigation wastes much more water than pressurized overhead or drip irrigation) and on irrigation scheduling, which is the method used to determine the amount of water to be applied to a crop and the timing for application. Since it determines the crop’s water use and influences its yield, irrigation scheduling has a remarkable effect on water use efficiency.

Irrigation scheduling is crucial in intensive agriculture, since under-irrigation generally results in reduced crop yield and quality. On the other hand, over-irrigation increases the nutrient requirements of the crop and its vulnerability to diseases, the energy costs for water pumping, water loss and environmental pollution due to the leaching of nutrients applied to the crop with conventional fertilization or fertigation (the technique of supplying fertilizers dissolved in the irrigation water). Thompson *et al.* [3] reported that the inadequate management of drip irrigation, which in many operations is still based on grower’s experience, is one of the reasons for nitrate leaching in greenhouse tomato production in Almeria, Spain (at present, the largest greenhouse area in the world).

The goal of an efficient irrigation program is to supply the crop with enough water while minimizing water waste due to deep percolation and runoff. Different approaches to irrigation scheduling have been developed, each having both advantages and disadvantages [4]. Innovative methods based on the direct monitoring of plant water relations have been also proposed for irrigation scheduling [4]. Although some companies have designed irrigation control devices exploiting micro-measurements of stem diameter, leaf thickness or stem sap flow, plant-based irrigation management is still in the research or development state and is scarcely employed in commercial operations [4].

The most widespread irrigation scheduling method is based on the determination of soil-water balance, which implies the estimation of crop evapotranspiration (ET_C_). Generally, ET_C_ is calculated combining the measurements of potential (or reference) evapotranspiration (ET_0_) through meteorological stations with crop coefficients [5]. The latter need regular updating by the farmer for each crop type and growing stage. One rather new approach is to obtain crop coefficients with satellite based radiation images, rather than by using time-costly manual field observations. Recently, D’Urso *et al.* [6] developed such a system within the framework of two European projects: Demeter (www.demeter-EC.net) and Pleiades (www.pleiades.es). Access to the satellite data has become much easier and faster due to recent development with web-based access and due to improvements of sensor spatial resolution and accuracy. D’Urso *et al.* [6] report that satellite remote sensing is a mature technique, suited to be transferred to practical application for on farm, down to the plot, irrigation management.

The other approach to irrigation scheduling entails the use of root zone sensor (RZS) to obtain soil moisture status and to replenish the water in growing medium to a preset level. In principle, this method by-passes the need to calculate ET_C_ and works for any crop, as long as the set-points for the irrigation controller are correctly chosen. So far, applications of RZS for irrigation management have been less common than those of the water balance method, but novel types of RZS, which are based on the measurement of soil dielectric properties, have opened new possibilities for irrigation scheduling and nowadays, after the doubts originated from the first attempts with gypsum blocks, the irrigation industry worldwide has recognized that RZS’ are valuable tools for modern smart water application technology in intensive agriculture.

In this review, the main features of RZS’ (for both soil moisture and salinity) designed to be connected to irrigation controller for commercial cultivations are identified and discussed. The paper is based on classical and more recent literature on soil moisture sensing technology and its application to irrigation scheduling.

The findings, also unpublished, of our recent experimental works conducted in the framework of national or international projects (see *Acknowledgements*) have been considered. These works concern mostly the outdoor cultivation of pot ornamentals, which is an important horticultural sector in Italy.

The main area for this kind of cultivation is located in Tuscany, around the town of Pistoia, definitely the most important centre in Europe for landscaping ornamentals [7]. In this area, nearly 1,400 ha, of approx. 4,500 ha of nurseries, are used for growing pot ornamentals, with an estimated yearly consumption of irrigation water (mostly, low salinity groundwater) of more than 10,000,000 m^3^/year [8]. The low cost of water has not led to a more efficient approach to water management. On the other hand, current legislation on water resources and the competition for water among agriculture, urban population and industries will affect the future development of “green industry” in this area, stimulating the search for more efficient solutions. Therefore, the design of a sustainable irrigation management system is one of the goals of RTD activities conducted by both public and private territorial entities (regional extension office, growers associations etc.)

The paper is agriculture-oriented and then it considers only those sensors that can be implemented in irrigation controllers, such as traditional water-filled tensiometers and the more recent dielectric sensors. Expensive and sophisticated RZS’ currently used by soil and plant scientists, such as neutron probes [9], are not addressed. The paper also discusses how these sensors may be integrated into wireless networks for computer-controlled irrigation and employed for the application of innovative irrigation strategies, such as deficit or dual-water (alternate use of different water sources) irrigation.

## 2. Soil Hydraulic Characteristics

Apart from the direct measurement of water content by thermo-gravimetric methods, soil moisture conditions can be assessed by the determination of the matric potential (ψ_m_) of pore water or the volumetric water content (θ), which is the volume of water in a certain volume of undisturbed soil. Recently, some sensors have been developed also to measure the soil salinity (namely, electrical conductivity o EC), as discussed later.

**Figure 1.**
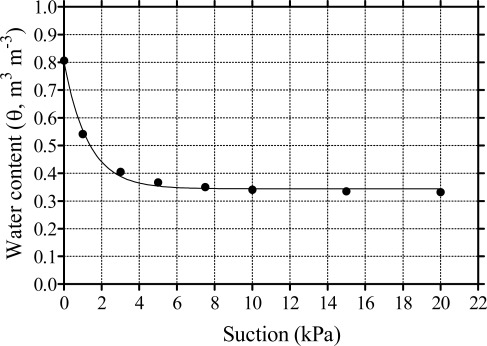
Water retention curve of a peat-pumice mixture, which is widely used for the outdoor production of ornamental nursery stocks in Italy. The determination was conducted in the laboratory following De Boodt’s method [11]. Seven different suctions were applied to the sample: 1.0, 3.0, 5.0, 7.5, 10.0, 15.0 and 20.0 kPa. The value of θ at saturation (ψ_m_ = 0) was estimated from the measurement of porosity (80.6%).

The term ψ_m_ describes the amount of energy which must be exerted to extract water from a porous medium such as soil or soilless substrate. Therefore, ψ_m_ corresponds to the suction required for the water uptake by the plant roots. Moisture tension represents the degree of such suction and, according to the terminology used in crop irrigation management, it is a positive number; the higher the number, the higher is the tension and the drier is the soil. The relationship between ψ_m_ and θ (also called water retention curve) depends on the nature of growing media and in some cases this relationship is not unique being different in drying and wetting cycle, in particular in fine-textured soils. The water retention curve is generally determined in the laboratory following standard methods, for instance: in soil, using Richard's pressure plate apparatus [10]; in soilless media (substrate) using De Boodt’s method [11]. An example of water retention curve for a soilless substrate is presented in Figure 1.

**Figure 2.**
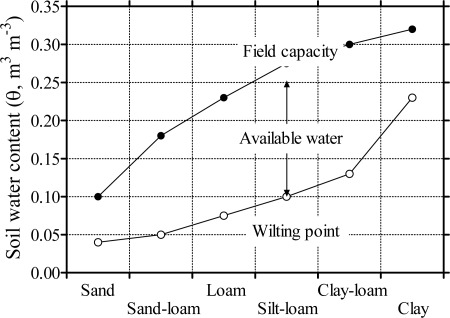
Water content at field capacity (θ_FC_) and at wilting point (θ_WP_), and available water (i.e., the difference between θ_FC_ and θ_WP_) in different types of soil.

Conventionally, the water retention curve may allow computation of a few parameters that, along with porosity and bulk density, are used to describe the water relations of a given soil sample. Field capacity (θ_FC_) is the water content in soil after it has been saturated (all pores are filled with water) and allowed to drain freely till the downward movement of water is terminated; wilting point (θ_WP_) is the minimal moisture the plant requires not to wilt. The physical definition of θ_FC_ and θ_WP_ is the bulk θ at ψ_m_ of −33 or −1,500 kPa, respectively [12]. The limitation of these determinations is evident, since many factors, also related to the plant, affect the actual values of θ_FC_ and θ_WP_. Nevertheless, these parameters serve as a practical measure of soil *w*ater holding capacity, also called available water. Soil available water is defined as the amount of water held between θ_FC_ and θ_WP_. Figure 2 illustrates how θ_FC_, θ_WP_ and available water changes as a function of soil texture. Readily available water is the fraction (generally, 50%) of available water that can be absorbed by the crop plant. The critical ψ_m_ at which many crops undergo water stress are in the order of magnitude of −100 kPa.

In other-than-soil media, such as peat, perlite, pumice and rockwool, which are largely used in greenhouses and nurseries, available water is the difference between the θ values determined at ψ_m_ of −1 (by definition, the ψ_m_ at container water capacity) and −10 kPa [11,13]. Depending on the nature of substrate, available water ranges from 0.07 to 0.35 m^3^ m^−3^ [13]; it tends to increase with the porosity and the bulk density of the material.

## 3. Soil Moisture Sensing Technology

### 3.1. Tensiometers

A tensiometer is a kind of artificial root that measures ψ_m_ in the growing medium. The tensiometer consists of a shaft filled with (distilled) degassed water with a porous ceramic cup at the end and a dial vacuum gauge or a pressure transducer at the top. The shaft is generally made of plastic due its low heat conduction and high resistance to corrosion. The ceramic cup has small air entry potentials in order to prevent de-saturation when subjected to negative potentials. The transducer can be connected to a data-logger for long-term monitoring, to a hand-held meter for spot measurements or to an irrigation controller. The tensiometer is buried at the desired depth in the rooting zone with the ceramic tip in close contact with the soil particles. The water in the tensiometer reaches equilibrium with the surrounding soil through the ceramic tip. When water is pulled out through the ceramic tip by drying soil, a tension is originated in the tube; when the soil is re-watered, the decrease in water potential gradient causes a reverse flow of water. As the soil goes through drying and wetting cycles as a result of ET_C_ and watering (by irrigation or rainfall), tension readings can be taken. The porosity of the ceramic tip influences the velocity of water flow from and into the instrument. When used in very dry conditions, the ceramic must be very fine and this slows down the tensiometer reaction to the changes in soil ψ_m_. Obviously, the responsiveness of tensiometers to soil moisture conditions determines the field of application. For instance, in container cultivation the irrigation lasts generally a few minutes instead of one or more hours as in soil culture; therefore, a quick response is required, especially when tensiometer is used to determine both the time and the duration of watering (see next section).

Tensiometers have many advantages that make them a competitive instrument for measuring soil moisture conditions [14]. They are accurate and not influenced by temperature and soil osmotic potential, since the salts move freely through the ceramic cup. Generally, tensiometers operate between 0 and 80°C. Below 0°C it is necessary to take some precautions due to ice formation, although some tensiometers can work even at −10 ° C (e.g., SKP 850; SDEC; the addresses of the companies mentioned in the text are reported at the end of our review).

Some companies focus on producing high grade hydraulic tensiometers with special features like high accuracy, high speed (small versions), ability to work over a large range of tensions and temperatures (dry end, no collapse of water column), easy use in the field (on the fly refillable), etc. These tensiometers are referred to as scientifically rated tensiometers; they have generally higher prices and are not commonly used by growers, but rather by soil and plant scientists.

On the Italian market, where to our knowledge no manufacturing company exists, the price of tensiometers ranges between € 150 – 300 and can reach as much as € 500, depending on the dimensions of the ceramic cup and shaft. Some tensiometers have small dimensions, allowing their utilisation in small pots. For example, Delta-T Devices Ltd. and Skye Instruments Ltd. produce, the SWT5 with a 7-cm shaft and the SKTM 650 with a 10-cm shaft, respectively.

However, tensiometers have also some disadvantages. They are quite fragile and must be operated carefully in order to avoid the formation of air bubbles in the shaft, which may result in temperature-dependent errors in reading. Moreover, they must be protected from frost and need regular maintenance, for instance to refill the water in the tube and to avoid the contamination by algae. Suitable hand-held vacuum pumps are generally supplied by tensiometer manufacturers to recharge tensiometers *in situ*.

When tensiometers are used in pot culture, some precautions are necessary in order to assure a good contact between the porous tip and the substrate, and to achieve a correct sensor positioning taking into account root distribution and the place of nozzle(s) in case of drip irrigation.

The main drawback of tensiometers, however, is the cavitation as the soil gets dry. The suction must not exceed the air entry value of the ceramic. The use of tensiometers may be difficult in soilless substrates [15], since their high porosity (> 80% against roughly 50% in soils) may result in a poor contact between the substrate and the ceramic tip. The typical operating range of water-filled tensiometers is between 0 and −85 kPa, with an accuracy of 0.1 – 1.0 kPa [14].

High capacity tensiometers have been developed in which the level of measurable suction is limited only to the air entry potential of the porous ceramic filter and the tensile strength of water [e.g., 16–18]. These instruments can work down to ψ_m_ as low as −2 MPa; however, their application has been limited by the lack of commercially-available devices and by the technical expertise needed for their correct use.

The porous-matrix sensor follows another approach to the measurement of ψ_m_ [e.g., 19,20]. This sensor uses porous ceramic with known water retention properties (pF-curve), which readily changes its degree of saturation to maintain equilibrium with the soil water. Soil ψ_m_ is determined indirectly from the measurements of the water content of the ceramic, for instance by means of dielectric sensor (see next section). Such sensors are commercially-available (e.g., equitensiometer; Delta-T Devices Ltd.). They can work in frozen and dry soils with ψ_m_ as low as −500 kPa. Moreover, they do not need maintenance for refilling or degassing likewise water-filled tensiometer.

Recently, in the FLOWAID project Whalley and colleagues have designed a new dielectric tensiometer that works over a much wider range of ψ_m_. A number of prototypes of this sensor have been tested both in the lab and in a greenhouse cucumber crop [21]. Good results have been achieved with these sensor prototypes that are able to measure soil ψ_m_ over the nominal range from −3 kPa to −250 kPa. These sensors might be commercially available in the next months.

A particular example of granular matric sensor is 229-L, which is manufactured by Campbell Scientific. In this sensor the moisture content in the ceramic is measured by the heat dissipation method. The sensor has a heating element and a thermocouple encased in the ceramic. The heating element warms up the ceramic and the thermocouple measures the temperature rise. The magnitude of the temperature rise varies according to the amount of water in the porous ceramic matrix. Each sensor must be calibrated according to soil type. This sensor requires a data-logger to control the current excitation module, to read the thermocouple outputs and to compute soil ψ_m_ (from −10 to −2,500 kPa).

### 3.2. Electrical resistance sensors

These sensors consist of a porous material in which electrodes are embedded to measure electrical resistance. When placed in the soil, the sensor tends to reach an equilibrium with soil water and the electrical resistance reading can converted to a calibrated reading of ψ_m_: the drier the soil, the greater the electrical resistance. Compared to tensiometers, the main advantage of electrical resistance sensors is their much wider measuring range [22].

The elements are covered with a synthetic membrane that protects the device from corrosion. The chalk element prevents the salinity influence on the measurements. Gypsum blocks are the oldest and simplest electrical resistance sensors [23]. They are inexpensive and maintenance-free, but less resistant and long-lived than other more advanced sensors. The gypsum material dissolves with time and therefore the sensor must be replaced every year. Moreover, their response to the change in soil moisture is slow, especially in soils close to θ_FC_. Generally, they work well in fine textured soil over a ψ_m_ range from −30 to −1,500 kPa approximately, while their use in coarse soil is quite difficult.

Watermark (Irrometer Company, Inc.) is an example of electrical resistance sensor [24]. The pore size of its matrix is larger than in gypsum blocks; this improves its sensitivity in wet soil and makes it possible to use this sensor in coarse soils and in soil moisture conditions close to θ_FC_. Other advantages of this sensor are the accuracy and the durability, albeit small calibration shifts may occur with time. However, the readings are affected by soil salinity due to fertilizers or irrigation water, although the sensors are normally supplied with built-in compensation for common salinity levels in the fields. Recently, in the FLOWAID project we tested Watermark sensors in container plants with poor results and we concluded that such a sensor is not suitable for monitoring the water status of soilless substrates (usually close to saturation) and for controlling irrigation in pot culture.

### 3.3. Dielectric sensors

In dielectric sensors an alternating electric field is induced into the surrounding media. By measuring voltages and currents induced by this field in the measuring rods, the total complex electrical impedance is obtained for the media. This impedance relates to the complex permittivity, which can be converted into soil θ and bulk EC, on the premises of known media calibration. The size and shape of the electric field depends largely upon the shape and size of the used electrodes for sensors. Furthermore, the penetration depth of the electric field is limited and decays rapidly the further away you get from the electrodes, as a reciprocal of the distance.

With this technique, only an average of the soil moisture in the measuring volume can be obtained. In non-homogeneous media or water content distributions one needs to take into account that the water closer to the electrodes contributes more to the average value. Especially in growing media like rockwool (widely used for fruit vegetables grown in greenhouses), where there is a large vertical gradient in θ, it is needed to calibrate the dielectric sensors for the specific placement in the media (vertical, horizontal, pin directions). For the 3-electrode version of the WET sensor (Delta-T Devices Ltd.; pin length 70 mm and distance of 2 × 16 mm), a measuring volume of about 220 cm^3^ was found when using a 99% electric field influence reaching out to about 2 cm from the electrodes [25]. Some sensors like Theta-probe (Delta-T Devices Ltd.) mimic a co-axial electrode design by having a single pin in the middle and three surrounding electrically connected outer pins. Their sensing volume is smaller and the readings are less susceptible for disturbances or variations in θ outside the pins. The smaller measuring volume for dielectric sensors might be a problem in non-heterogeneous soils. Here, one wants to obtain an average reading over a large volume, to get a more representative reading with only a single sensor. Several sensor concepts try to enlarge their volume by making larger pin concept, for instance AquaFlex (Streat Instruments Ltd).

There are different types of dielectric sensors depending on the output signal used for estimating θ, based on Time Domain Reflectometry (TDR), Frequency Domain (FD) (Reflectometry and Capacitance), Time Domain Transmission (TDT), Amplitude Domain Reflectometry (ADR) and Phase Transmission sensors. All these sensors differ in terms of use and maintenance, calibration requirements, accuracy and price [26]. While TDR uses a pulsed excitation wave, FD uses a fixed frequency sine wave to measure the soil impedance. The precision and the accuracy of TDR and FD sensors depend on the wave form interpretation methods used in software [27]. Most of them are based on the relationship between θ and dielectric constant (permittivity) of soil according to the Topp’s equation [28]. The latter can be valid for the soils with low clay content and a bulk density between 1.3 and 1.5 kg L^−1^ [29]. For other kinds of soil special calibration is generally required [30].

For many years TDR has been the only principle used to measure soil θ. Expensive equipment is necessary for this kind of measurement; therefore, TDR technology is considered less suitable for irrigation control in commercial crops, although very recently some authors [31] reported on the application of TDR sensor for irrigation scheduling in nursery and greenhouse industry.

FD-sensors for soil moisture (e.g., Theta-probe, Delta-T Devices Ltd.) came onto the market more recently. The main advantage of FD sensors is the immediate and accurate reading, thus they are quite suitable for automated irrigation control. However, there are some drawbacks. For instance, they are affected by soil salinity, which attenuates the signal. There is also general consensus that FD sensors must be calibrated more frequently than TDR sensors. Using a frequency of at least 1 GHz can minimize this problem [32]. Soil porosity and temperature may also influence sensor readings, especially when frequencies lower than 20 MHz are used [23]. In the soil top layer, wide temperature fluctuations occur during the day and the growing season, therefore the measurement of temperature must be incorporated in the sensor in order to allow on-line correction. Likewise any other RZS, dielectric sensors must be placed in strict contact with soil, since air gaps may result in erroneous measurements.

In a series of laboratory or greenhouse experiments, we have evaluated a few dielectric sensors such as Theta Probe ML2X and SM200 (both from Delta-T Devices Ltd.). Generally, these sensors consist of two or more electrodes separated by a plastic dielectric and positioned inside a cylinder; a plastic access tube covers all these elements. Obviously, the design may change according to the specific application. The measuring volume depends on the distance between the two conductors and on their length. In common soils, Theta Probe is characterized by a 5% accuracy using the supplied calibration, but it is possible to achieve 1% accuracy after calibration to a specific soil. This sensor seems quite suitable for small-volume pots and containers such as plug trays, which are largely used in greenhouse and nursery crops. It can work up at temperature as high as 70 °C, although the accuracy decreased above 40 °C, always considering a calibration to a specific soil.

Some experiments were carried out on sandy soils and coconut fibre to verify the applicability of Theta Probe ML2X in some growing media widely used in ornamental crops [32]. The repeatability of measurements was good, both on all tested sandy soils tested and on coconut fibre as well. About the salinity effects on data acquisition, in a 0.7 – 0.4% dS m^−1^ range, the salinity of the nutritive solution on coconut fibre substrate affected sensor reading with an error of ± 3 – 4%. However, this error was unimportant and the use of Theta Probe ML2x to control irrigation of plants grown in coconut fibres did not affect plant growth. Farina and Bacci [32] also observed that the temperature did not influence the repeatability of sensor readings. Theta Probe revealed some limitations in presence of lack of homogeneity in soil characteristics or when an incorrect installation occurs. In fact, it’s strongly recommended to insert sensors into the soil without torsions that could lead to a poor contact between sensor rods and soil particles.

SM200 sensor, which is more recent than Theta Probe, is suitable for many types of soil and substrates. Moreover, it is barely affected by soil salinity and can be used at extreme temperatures, since the operating range is between −20 and +60 °C. Measuring accuracy is ±0.3% for θ from 0 to 0.50 m^3^ m^−3^; as a function of temperature, the accuracy is ±0.07% at 20 °C and 0.13% in the 20 – 60 °C range.

In the recent past, a new generation of dielectric sensors has been developed to measure both θ and the salinity (namely, EC) of growing media, thus providing the possibility to control the fertilisation as well, for example by adjusting the concentration of the fertigation water. Currently there are two sensors on the market: the WET (Delta-T Devices Ltd.) and the 5TE (Decagon Devices Inc.).

The WET is a frequency domain dielectric sensor that was originally designed and produced by IMAG-DLO Wageningen (The Netherlands) [33] and is now manufactured and sold by Delta T-Devices Ltd. at a cost of € 600 – 900. It is a 3-pin sensor, with the main advantage that it measures water content, EC and temperature in an all-in-one sensor. The temperature readings are used to correct the θ and EC variations with temperature. The sensor has a high repeatability once inserted into a growing medium and left untouched (1%). However, to obtain good absolute readings, the sensor needs a specific calibration for the type of growing medium. In its simple form, one may use the Topp’s curve [28], which was made for TDR measurement at 150 MHz. Since the WET works at 20 MHz, it is much more susceptible to soil texture and the manufacturer advises to use their standard curves for sand, clay, mineral soils etc., or to use a customer-defined calibration curve. The WET is used for the agricultural market especially for irrigation and fertigation control, mostly in greenhouses horticulture. In this market, and as a derivative of the original WET, it is sold by Grodan B.V. under the name of WCM. WCM can be used to control the root zone. Stradiot [34] even reports that by maintaining differences between day and night moisture rockwool slabs it is possible to steer plants towards vegetative or generative growth.

The WET was originally designed to be used in soil, where normally EC is lower then 2 dS m^−1^. In horticultural growing media, however, EC may be as high as 10 dS m^−1^. For this reason, Grodan has modified the original design and calibrates their sensors up to 10 dS m^−1^, and Delta-T Devices Ltd. supplies extended calibration curves up to 5 dS m^−1^. In highly-saline soils, the accuracy of the standard WET is not warranted; Bouksila [35], for instance, advises to use a soil specific calibration here as well.

The WET measures bulk EC, which is the total EC of the growing media containing water, nutrients and air. However, generally growers are not interested in bulk EC, but rather in pore water EC (EC_pw_), which is the EC in the water extracted from the growing media, for instance by using suction cup or by performing a 1 : 2 water extract [36].

Hilhorst [37] developed a derivative of the WET, which has an easy-to-handle design for taking rapid and multiple readings in container plants. It has a single insertion pin containing both electrodes in the tip, and measures EC_pw_ directly by using a special model. Delta-T Devices Ltd. brought this probe onto the market (Sigma-Probe), but due to mechanical problems, and especially the limited range over which the EC_pw_ model was valid, it was taken out of the market. Since then, several researches reported about modifications of the Hilhorst-model of EC_pw_ to make it possible to use the WET for direct measurement of EC_pw_ [e.g., 38,39].

In Italy, especially in container grown crops, mixtures of several materials such as peat, perlite and pumice (of volcanic origin) are being used as growing media. Regalado *et al.* [40] reported about potential problems with the calibration of the WET sensor in volcanic soils, due to the biased determination of θ. Therefore, they proposed to use an empirical calibration for the water content based upon soil bulk density, and an estimate of the sensor’s effective frequency. Recently, Incrocci *et al.* [39] conducted a study on the calibration of the WET using different types of growing media (perlite, pumice, peat and mixtures of these materials). They concluded that: i) θ calibration was faintly dependent on the type of substrate and only slightly affected by the salinity of irrigation water; ii) at least in the peat-pumice mixture, the linear regression between bulk EC and EC_pw_ was markedly affected by θ, the slope decreasing with increasing θ. An equation differing from the one published by Hilhorst [37] was proposed to estimate EC_pw_ from the WET readings of permittivity, bulk EC and temperature.

The 5TE sensor is a low-power sensor that measures soil θ, temperature and bulk EC. It is very new on the market (it appeared in 2007) and costs about half the price of the standard WET, due to its simpler housing design, based upon a moulded and combined electrode-printed circuit board design. It has an RS232 output, which makes it necessary to use an external micro-computer to handle the data, and makes it less suitable to connect it to standard data loggers. The 5TE outputs just three simple values for permittivity, EC and temperature, which makes interfacing quiet simple. The WET-sensor has a similar serial interface, but decoding its data requires a more complex algorithm.

The 5TE uses 3.6 – 15 VDC at 10 mA during 150 ms measurement. Its operating temperature range is between −40 and 50 °C and it has small dimensions. θ values are obtained by using the FD technology at about 70 MHz (based upon the previous ECH_2_O-probe), and by utilizing a higher frequency than the WET. Moreover, the 5TE has minimal salinity and textural effects. It has a resolution of 0.08% from 0 to 0.50 m^3^ m^−3^ θ, and an accuracy (when using a medium specific calibration) of ± 1 – 2% in any porous medium with EC < 10 dS m^−1^. EC is obtained using a separate low-frequency EC technique based upon the EC-5 probe, and two stainless steel screws which makes the sensor more robust. The operating range is 0 – 23 dS m^−1^ (bulk EC), with a resolution of 0.01 dS m^−1^ from 0 to 7 dS m^−1^, and 0.05 dS m^−1^ from 7 to 23 dS/m. Accuracy is ±10% for 0 – 7 dS m^−1^; user calibration is needed above 7 dS m^−1^. For a number of mineral soils, a single θ calibration curve seems suitable. The probe uncertainty increases with increasing θ, and at higher EC values the probe signal is attenuated. Bogena *et al.* [41] tested the previous EC-5 probe and concluded that temperature and EC effects on the sensor reading should be corrected, using appropriate correction functions. However, they, as well as Kizito *et al.* [42], report that the family of ECH_2_O sensors (EC-5 and ECH_2_O-TE) is suited for large RZS networks because of their low-cost and limited needs for calibration.

## 4. Irrigation Controllers

Most of the off-the-shelf sensors are equipped only with manual or digital readers, able to make many elaborations but without any device to automatically activation the irrigation process. However, all sensors can be integrated in a dedicated system for automatic irrigation.

The simplest irrigation controller based on RZS is represented by a switching tensiometer. There are two main systems available commercially. The first consists in an electronic switch that can be mounted on the vacuum gauge dial of the tensiometer and set to start irrigation at pre-set values of soil tension. This switch normally operates with AC current flow, but it’s possible to order direct current (DC) systems. The vacuum gauge is equipped with a magnet and a magnetic pick-up switch. When the pre-set soil tension is reached, the switch closes and activates the irrigation pump, which distributes a pre-fixed amount of water. The second type of irrigation controller uses a pressure transducer instead of a vacuum gauge. The pressure transducer measures any variation in soil tension and modifies the electric current flow accordingly; the reading is continuous. More advanced irrigation controllers are, for instance, the GP1 irrigation system manufactured by Delta-T Devices Ltd. and the prototype developed by Bacci *et al.* [43].

With GP1, Delta-T Devices Ltd. also allows the possibility of upgrading an existing controller (central controller, residential or commercial timer) by adding intelligence to it. GP1 optimizes watering using both simple and complex irrigation algorithm based on multiple parameters, including soil moisture and weather data, allowing multiple irrigation ‘start-and-stop’ conditions.

The prototype developed by Bacci *et al.* [43] is based on a programmable data-logger (Tattletale 5F, Onset Computer Corporation), to which up to eight tensiometers and other sensors (e.g., thermometer, hygrometer, anemometer, net radiometer etc.) can be connected. SKTM 650 (Skye Instruments Ltd.) tensiometers are used in consideration of their low price, accuracy and small dimensions. Obviously, other RZS’ could be employed. The current version of this device can control four electro-valves. The built-in software allows the setting of ψ_m_ threshold to active irrigation and the management of the irrigation cycle (time, dose, etc.). Each tensiometer can be managed independently or the irrigation activation can be driven by the average reading of two or more tensiometers. The software implements an algorithm to calculate ET_0_ according to CIMIS equation [44]. Hourly ET_0_ values are used to check the irrigation method based on ψ_m_ measurements according to an integrated system described in Bacci *et al.* [43]. The new version of the system under development will be provided of a 32 BIT CPU with: eight analogic channels for the acquisition of different parameters (micrometeorological data, soil water status etc.); eight I/O digital counters for water meters measuring the volumes of both irrigation and drainage water; electro-valves control. System expansion will be assured by RS-485 port that allows the connection of S-bus modules of two types: analogical (signal range 0 – 5V; 0 – 20 mA) and digital (relays control). The S-bus interface allows the management of actuators and sensory by means of an only bus to four wires: two wires to power supply and two wires to digital signal (informative content). The bus connects the different modules in cascade, eliminating the problems associated to wiring and the interference on the sensor signal.

### 4.1. Sensor positioning

For the growers, a complete irrigation control system must be cost effective and user-friendly. The sensors used for monitoring soil water content must be inexpensive and the minimum number of sensors needed for a given irrigation plot must be clearly defined. Moreover, the sensor must be easily removed and reinstalled, as in many farms (for instance, in greenhouses and nurseries) there are several crops over the year. Knowing where and how many sensors are positioned in the crop is therefore crucial.

A single RZS may be not enough due to possible malfunctioning and, more importantly, because it monitors only a limited volume (sensor cup has a small sphere of action) and area of the cultivation. Large variability in the root zone moisture may occur as results of the heterogeneity in both soil/substrate characteristics and plant water relations. Also under relatively uniform conditions as those occurring in greenhouses and nurseries, there is large variability in the ET_C_ of individual plants, and this may result in important differences in the soil dehydration rate among different zones in the irrigated area. For instance, in greenhouses the plants close to lateral windows and/or to heating systems tend to transpire much more, compared to plants growing in other locations.

In an open-air nursery at CESPEVI in Pistoia (Italy), Bacci *et al.* [45] determined the readings of 12 identical tensiometers each placed in different pots filled with a peat-pumice (1:1, v:v) mix. The pots were located within 0.5 m each from other. The variability ranged between 17% for container water capacity and 21% for ψ_m_ −7.5 kPa (the irrigation set-point fixed for the experiment). Thus, more RZS’ are needed in the irrigation area in order to obtain significant mean values of soil water status (also by appropriate statistical treatment of data) and to overcome the potential problems due to sensor breakdown.

Sensor position inside the growing medium is another important issue. In field crops, it is generally recommended that RZS’ be installed in pairs, one at one-third and the other at two-thirds of the rooting depth. In pot plants, the root system has a propensity to develop in the bottom half of the container with very few roots in the top substrate. Moreover, irrigation frequency is very high (up to several times a day) due to the limited water buffer capacity of the pot as a whole. Under these conditions, in-between successive irrigation events there are minimal variations of the overall θ, which remains close to θ_FC_. However, there is a large vertical gradient in θ: at the bottom, the substrate remains nearly saturated and shows minimal variations in θ, whereas the top layer desiccates rapidly after irrigation due to the gravitational water flow, thus showing wide oscillations in moisture. Therefore, RZS’ must be placed in an intermediate position. In plants cultivated in pots with a volume ranging from approximately 1.5 to 9.0 L (pot diameter of 12 or 18 cm, respectively), Bacci and colleagues [46,47] found that the best position is at one-third of the pot height from the top and at 3 – 4 cm from the pot edge. It was also found that, when drip irrigation is used, the sensor should be inserted in the substrate at a 90° angle towards the nozzle.

## 5. Irrigation Strategies Based on RZS

Many research papers have documented that the schedule of irrigation by means of RZS may significantly reduce the water use of many field and greenhouse crops (cultivated in both soil or soilless media) without any important effects on crop productivity. A few examples are those published very recently by Zotarelli *et al.* (field tomato) [48], Tuzel at al. (greenhouse cucumber) [21] and Pardossi *et al.* (outdoor pot ornamentals) [8].

When RZS’ are applied to schedule irrigation, the controller uses the data from the sensing point(s) in the root zone to determine the moment and duration of watering. The operating principle is that, whenever a pre-set threshold (‘start’ value) of θ or ψ_m_ is reached, the valves of the irrigation pipelines are opened till the ‘stop’ value (generally, close to θ_FC_) is reached. In addition to ‘start’ and ‘stop’ thresholds, RZS-based irrigation controllers generally require other inputs from the user, such as minimum and maximum duration of watering, and maximum elapsed time without irrigation in consideration of possible sensor failure. Several devices (e.g., GP1, Delta-T Devices Ltd.) are commercially available to provide a ‘start-and-stop’ irrigation control based on two or more RZS’ buried in and underneath the root-zone. This set of sensors can monitor the movement of the water into the deeper layers and minimize percolation losses.

The θ or ψ_m_ ‘start’ value depends on crop species and developmental stage, soil characteristics and environmental conditions as well. For ψ_m_, the typical range is between −20 and −70 kPa in soil (depending on soil texture) or between −4 and −10 kPa in soilless substrates [47]. By determining the divergence in leaf water potential between well-watered and un-watered plants, Thompson *et al.* [49] identified the ψ_m_ threshold for pepper, melon and tomato grown under the typical greenhouse conditions of south-eastern coast of Spain: they were −58 kPa for pepper, −35 kPa for melon and −38 or −58 kPa for tomato grown, respectively, in spring or in winter. Therefore, optimal ‘start’ ψ_m_ value may decreased under lower ET conditions, at least in some crop species.

### 5.1. Deficit irrigation

RZS may be valuable tool for the implementation of regulated deficit irrigation, which aims to control the vegetative vigour of crop plants and improve produce quality [50]. For instance, regulated deficit irrigation is largely used for wine grapes in regions where this crop is regularly irrigated, such as California and Australia. In red grape varieties, a moderate water stress imposed a few weeks after fruit set and maintained midway through berry formation may enhance the maturity of berries and improve the quality of wine. For best results, it is reported that the average soil ψ_m_ should be kept at −150 to −200 kPa in sandy soil, around −250 kPa in loamy soil and at −300 to −400 kPa in clay soil [51]. Therefore, a successful application of deficit irrigation needs the determination of soil water status and, in consideration of the range of ψ_m_ previously reported, tensiometer are of little value and dielectric or granular matrix sensor must be used.

An example of deficit irrigation regulated by RZS is the work conducted very recently by Tuzel *et al.* [21]. In a greenhouse cucumber crop at Izmir (Turkey), they used GP1 controllers with WET sensors buried at 20-cm depth to control full irrigation or two deficit irrigation regimes, as compared to grower’s practice (experience-based scheduling). Water was applied when θ reached 0.25, 0.22 and 0.18 m^3^ m^−3^, respectively, in Full, Deficit 1 and Deficit 2 irrigation treatments, which corresponded to a depletion of available water in the root zone of 20, 40 and 60%, respectively. It was found that RZS-based irrigation resulted in no noticeable percolation loss or over-irrigation in contrast to the traditional farm practice. Moreover, deficit irrigation increased crop water use efficiency by 40 – 42% and 27 – 28% compared to grower’s schedule and full irrigation, respectively.

### 5.2. Zero-runoff irrigation of container crops

The use of RZS seems very promising in the outdoor cultivation of pot ornamentals. These crops are generally over-irrigated for a series of reasons, such as poor irrigation scheduling (typically based on growers’ experience and the use of simple timers) and the plant sensitivity to salinity, which inevitably increases the leaching fraction, which is the fraction of applied water that is drained out from the pot bottom drained water. A leaching fraction of 25 – 30% is generally necessary to prevent the salinity build-up in pot plants, especially when fertigation and/or saline water are used by the growers. Moreover, in many nurseries, at least in the area of Pistoia, there is the practice to place different plant species in the same cultivation plot, which results in the tendency to regulate irrigation on the basis of the most water demanding crop.

In a work conducted recently at CESPEVI under the typical growing conditions of commercial nurseries (in particular, growing different plant species in the same plot), Pardossi *et al.* [8] compared timer and tensiometer control of fertigation of pot ornamentals. In the latter treatment, the irrigation was controlled by means of tensiometer placed in one pot hosting a plant (the sentinel) of *Prunus laurocerasus*, the species with the average ET_C_ among the ones under investigation; the others were *Forsythia intermedia*, *Photinia x fraseri,* and *Viburnum tinus*.

Compared to timers, the use of tensiometers decreased the water application by 23% and the leaching of nutrients with drainage water by 31%, thus alleviating the contamination (with nitrates, for instance) of ground and surface water. No differences between the two scheduling methods were observed in terms of plant growth and market value, as assessed at the end of the growing season. The reduction of water application allowed by RZS resulted from a lower frequency of irrigation compared to timer-based control, with minimal differences between the two methods in terms of leaching fraction.

**Figure 3.**
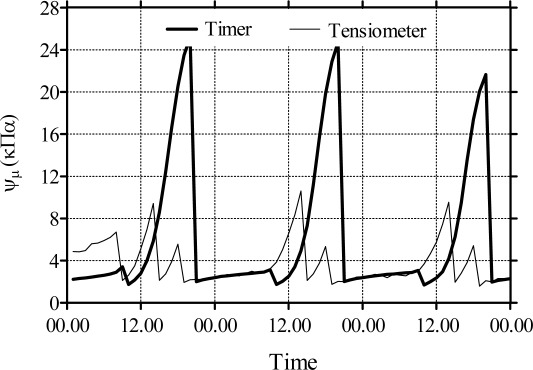
Changes in the substrate ψ_m_ in pots with *Hypericum hidcote* plant as recorded in three following days in July 2004. The pots were irrigated using a timer (twice per day, at 09:00 and 19:30; thick line) or a tensiometer (thin line) with a triggering threshold of −6.0 kPa and four time windows (07:00 – 08:00; 13:00 – 14:00; 17:00 – 18:00; 21:00 – 22:00) in order to simulate the commercial operational conditions in the nurseries in Pistoia (Italy) district.

Very similar results were found in another experiment carried out at CESPEVI by Bacci *et al.* [43] with container cultivation of *Hypericum hidcote*. They observed that, compared to timer-based irrigation scheduling, the tensiometer maintained a more favourable substrate water status to plant growth in periods of peak ET_C_, as suggested by Figure 3 where the data of ψ_m_ recorded during three following days in July 2004 are shown. In timer treatment, ψ_m_ decreased down to approximately −25 kPa, while in tensiometer-controlled culture ψ_m_ remained below −10 kPa in reasons of best water distribution during the day, notwithstanding the irrigation was permitted only in four preset time windows, according to the local growers’ practice. In soilless substrates growth reductions can occur at ψ_m_ as low as −16 kPa [e.g., 52,53].

Further improvement of irrigation efficiency in container crops may be attained by means of ‘start-and-stop’ control system. Such a system should maintain ψ_m_ between −8 to −10 kPa and −2 to −3.0 kPa, which correspond to θ values of, respectively, 0.30 – 035 and 0.45 – 0.55 m^3^ m^−3^, depending on the nature of growing medium. In this range of soil ψ_m_, the pot water content remains lower than θ_FC_ and the loss of water by drainage is minimized. This system, known as ‘zero-runoff’ irrigation, may be achieved also by the *a priori* determination of the water dose needed to increase substrate θ from the ‘start’ value to the value close to water capacity. Obviously, such a dose depends on both pot volume and the hydraulic properties of the growing media.

Following this approach and using greenhouse-grown pot geranium as plant model, in 2006 we conducted a trial at the University of Pisa to compare two scheduling methods (timer vs. tensiometer) in open drip irrigation system (L. Bacci and L. Incrocci, unpublished). Rooted cuttings of geranium *(Pelargonium peltatum*) were transplanted in pots with volume of 1.5 L and height of 13 cm, filled a peat-perlite mixture; plant density was approximately 8 plant m^−2^. Every day at a fixed time (08:00 a.m.) or whenever the tensiometer (SKTM 650) reading reached the threshold value (−6 kPa), a pre-established water dose was applied to each pot by means of self-compensating drippers (discharge rate of 2 L h^−1^). In timer treatment, the water dose ranged between 1.2 to 1.8 L m^−2^ according to the growth stage and the climate inside the greenhouse, that is the dose tended to increase during the cultivation; actually, in timer treatment we followed the growers’ practice. Conversely, in tensiometer-based irrigation, a fixed water volume of 1.8 L m^−2^ was established preliminarily in order to restore the full water container capacity with a leaching fraction not higher than 5%. This quantity was divided in three doses, four minutes apart. No differences between the two methods were observed regarding crop ET_C_, growth and commercial value; however, at the end of cultivation in tensiometer treatment water runoff was less than 1.0 L m^−2^ (approximately 2% of total applied water) against 14.3 L m^−2^ (23% of applied water) in the one scheduling method.

Nevertheless, the application of zero-runoff irrigation in pot cultivation has some difficulties. Firstly, it requires the use of raw water with low salinity; otherwise there is a progressive salinity build-up in the root zone, which results in plant growth reduction. Moreover, it is necessary to install pressure-compensating nozzles with uniform and low discharge rate, in order to synchronize the substrate moistening and the response of RZS. Too fast irrigation may lead to over-watering before RZS senses the ‘stop’ conditions in the measuring volume (generally, a small fraction of total container volume). Finally, the sentinel pot(s) does/do not provide a perfect representation of water needs of all plants in the plot, therefore extra water is required to ensure that the driest pots in the irrigated area (because of higher ET_C_ and/or lower discharge rate than expected) will receive the necessary dose. As matter of fact, real zero-runoff irrigation in container culture can be achieved only through the recovery of drainage water (closed system) [8]; in free-drain (open) culture the goal is to reduce leaching fraction as much as possible, say, below 10 – 15%.

### 5.3. Fertigation and dual water irrigation

The availability of sensors (WET and 5TE) that are capable of also monitoring the soil salinity has opened new possibilities for the automatic control of fertigation and/or the application of a dual-water irrigation scheme. In the FLOWAID project, in cooperation with Spagnol Greenhouse Technologies we developed a prototype of fertigation device, which is able to change the nutrient content (thus, EC) of the fertigation water as well as the source of raw water based on the measurements of the EC_pw_ of the growing medium (peat-pumice mixture) by means of WET sensors.

The prototype was tested at CESPEVI in the spring and summer of 2008 in an experiment with pot ornamental plants (the same used in the trial described in paragraph 5.2), where we simulated the availability of saline water (EC = 1.50 dS m^−1^) (L. Incrocci *et al.*, unpublished). Saline water might be the kind supplied, for instance, by an urban wastewater treatment plant. A project is underway in Pistoia district for an irrigation network using the treated wastewater from the city.

**Figure 4.**
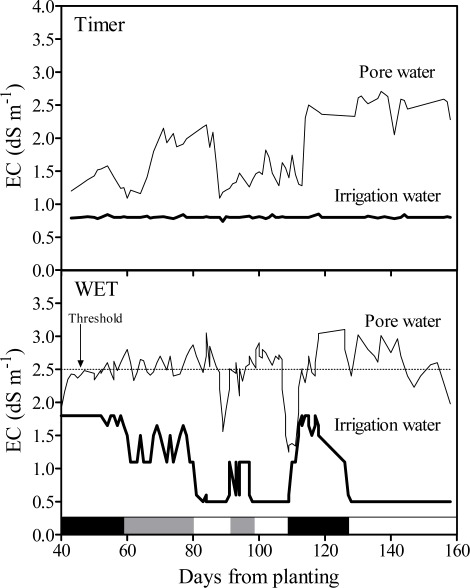
Changes in the salinity (EC) of the pore water (EC_pw;_, thin line) in the growing medium (peat-pumice mixture) and of the nutrient solution (thick line) fed to pot ornamentals grown at CESPEVI (Pistoia) in the spring-summer of 2008. Two irrigation regimes differing for scheduling method and the source of raw water were compared: : 1) timer control with nutrient solution (EC = 0.80 dS m^−1^) prepared using low-salinity groundwater (EC = 0.50 dS m^−1^); 2) WET scheduling and two sources of water (groundwater or saline water with EC = 1.50 dS m^−1^) to prepare the nutrient solution with a maximum EC of 1.80 dS m^−1^. In the latter, the nutrient solution EC was modulated by changing the rate of fertiliser addition and the water source in order to maintain the EC_pw_ below 2.50 dS m^−1^. In the lower graph, the bars close to the abscissa indicate the source of irrigation water: saline water (black), groundwater (white) or mixed water (grey).

Two fertigation regimes were compared: i) timer (one or two irrigations per day) control with nutrient solution (EC = 0.80 dS m^−1^) prepared using groundwater (EC = 0.50 dS m^−1^); WET scheduling with a θ threshold of 0.52 m^3^ m^−3^ and two sources of water (groundwater and/or saline water) to prepare a nutrient solution with a maximum EC of 1.80 dS m^−1^. The prototype modulated the EC of the nutrient solution by changing the rate of fertiliser addition as well as the sources of water, in order to maintain the EC_pw_ below 2.50 dS m^−1^, the maximum tolerable salinity level for the plants under investigation. During the growing season the prototype used saline water, fresh water or both (at variable ratio), as shown in Figure 4. The irrigation regime did not influence plant growth. However, compared to the timer, the WET-based scheduling reduced the seasonal water consumption by 35% (499 vs 325 L m^−2^), which was provided by saline water by 45%; therefore, in the WET treatment the consumption of groundwater was 36% of the one recorded in the timer treatment.

### 5.4. Integrated scheduling method

Irrigation timing and water dosage are the key aspects of watering scheduling. Available equipment and specially soil moisture monitoring devices, provide all the information required for irrigation management, but frequently lack in representativeness and reliability. To solve this problem, ET computing and soil moisture monitoring methods should be integrated to improve automatic irrigation system performances in field and in pots [54,55] and to guarantee system functioning in case of sensor damaging [56]. Unfortunately, the application of climate-based methods to pot plants requires a different approach to crop coefficient computing [e.g., 43,57].

Norrie *et al.* [58] developed an automatic irrigation system that integrated both electronic tensiometers and weather station to estimated ET_C_ with Penman-Monteith equation [5]. This system was tested in a greenhouse soilless culture (peat bag) of tomato. The information about ET_C_ was used to define the ‘start’ tension threshold as well as the concentration (EC) of fertigation water: during periods of intense ET_C_ the irrigation was more frequent (i.e., the trigger set-point was less negative) and the EC was reduced with respect to periods with low ET_C_.

More recently, Bacci *et al.* [43] suggested the use of tensiometer readings for the estimation of crop coefficient in potted plants using the CIMIS equation [44] for the assessment of ET_0_. In this way, after a short period of calibration for different ornamental plants grown in containers, the system can work autonomously, controlling the functioning of tensiometer by means of an indirect estimation of ET_C_.

## 6. ICT Technology for Irrigation Management

### 6.1. Decision support system

Due to soil spatial variability, many sensors are needed in each irrigation plot or for every group of sprinklers or drippers in the field, thus resulting in high investment and maintenance costs, especially when wired sensors are being used. Gathering real-time, fine-grained data is critical for success, but it is not possible with current wired data-loggers which are both expensive and not able to react to significant events (e.g., to increase sensing rate during a rain storm). Nowadays wireless communication is a simple and low-cost technology thanks to the wide diffusion of cell phones, ISM, GSM, WI-FI devices and SMS bi-directional delivery, satellites and the Internet [59]. Moreover, it enables to communicate quickly and store a great amount of information helpful in water budgeting and irrigation scheduling.

**Figure 5.**
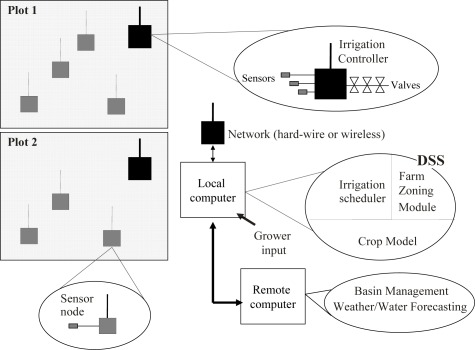
Management system for farm level irrigation.

The combination of the new technology with data processing algorithms and models could be used to develop decision support system (DSS) with positive effects for a sustainable and efficient use of natural resources in agricultural production and enabling the decision maker to optimise production and profit through cost reduction. An example of DSS is the FLOW-AID system [60]. This system consists of an array of irrigation controllers distributed over the farm zones to be irrigated (Figure 5). The controllers are connected via a wireless communication link to a local computer, which retrieves regularly the data from RZS’ and weather station, and updates the irrigation scheduling programs running autonomously in the controllers. A DSS running on the local computer and partly on a remote computer (connected via the Internet) assists the grower in the optimization of the scheduler programs for the irrigation controllers on a long-term as well as on a short-term basis. For example, on a long term the DSS provides the grower with an optimized crop planting plan, a set of irrigation scheduling tasks and an estimate of annual water use per each irrigation plot. On a short term, and based on actual crop status, weather and, possibly the weather forecast, the DSS irrigation scheduler module predicts crop water demand and suggests the optimal scheduling strategy for each plot, including the source of water if different types of water are available (well, reservoir, recycled water, irrigation network etc.). The selected irrigation tasks, including parameter settings, are then downloaded into the remote irrigation controllers. The irrigation scheduler is run on a day-to-day basis (or hour-to hour, for instance in container cultivation) and, if needed, the individual controllers are reprogrammed. Each irrigation plot has an individual controller node, and local or remote sensors are added to it as needed by the application, either via hard-wire or a wireless link. A typical node has the tasks to monitor soil water status and other data (for instance, soil and air temperature), and to open and close multiple valves. Once programmed by the irrigation scheduler, the controller keeps on running its tasks autonomously, until it is re-programmed or stopped by the local computer. This makes the irrigation of individual plots fairly safe as it does not depend on real-time communication with a remote computer.

### 6.2. Wireless sensor networks

Wireless sensor networks are a new technology that promises fine grain monitoring in time and space, and at a lower cost, than is currently possible [60,61]. Wireless sensor networks for irrigation management are presently offered by several companies (e.g., Crossbow Technologies, Inc.; Netafim Ltd.; Delta-T Devices Ltd.; Decagon Devices, Inc.). However, these systems are still quite expensive and have high-energy requirements. According to several authors [e.g., 62,63], at the current stage of development wireless sensor networks are not reliable and affordable enough for wide commercial application, even if very recently we [60] and other authors [64,65] have presented a new low-cost multi-hop wireless sensor network monitoring real-time substrate moisture, temperature and EC with good results in the control of irrigation and nutrient applications.

According to some tests proposed by Cardell-Oliver *et al.* [66], wireless sensor networks have great potential to provide dynamic, real-time data about monitored variables of a landscape enabling scientists to measure properties that have not previously been observable. However, to ensure effective data gathering by sensor networks for monitoring remote outdoor environments, the following problems remain: reactivity, the ability of the network to react to its environment and provide only relevant data to users; robustness, the ability of network nodes to function correctly in harsh outdoor environments; network lifetime, maximizing the length of time the network is able to deliver data before batteries are exhausted.

In the FLOWAID project, Balendonck *et al.* [60] experimented in container grown ornamentals with a self-made low-cost wireless sensor network (hybrid mesh network design) based upon the T-node system from SOWNET Technologies B.V. combined with an SM200 (Delta-T Devices Ltd.) sensor. The T-Nodes are the ones originally developed by TNO Defence, Security and Safety (The Netherlands) for wireless sensor networks. They combine a microcontroller for local processing, a radio for digital communications and I/O abilities to interface with sensors, actuators and external systems. They use an 868 MHz FSK transceiver with ranges up to 120 meters in free space and 40 meter indoors. They have power consumption (at 3V) of 18 mA (TX) and 13 mA (RX), and 20 μA in sleep mode; moreover, they have a self-organizing ultra low power multi-hop protocol and an ultra-compact design. Six sensor nodes and three repeaters were built and evaluated during five months in spring-summer 2007 in semi-commercial nursery operational environment at CESPEVI. This work continued during summer 2008 with an improved design: a new antenna design for higher signal strength; housing with a double wall; a construction for protection of the electronics against high temperatures. The software in the firmware of the wireless nodes was changed to optimize power use. In the current configuration, such a system costs about € 700 per node, including an SM200 sensor (about € 250). Functional tests showed that a maximum range of about 100 meters between node and repeater is feasible under line-of-sight conditions. Long term experiments at CESPEVI showed that a range of 60 m can be obtained. However, some short and not-so-short lasting node and sensor failures occurred either due to non-optimal mechanical construction of the housing, condensation, and unforeseen external events (theft). Even if in the second year there was much progress with the communication abilities of the SOWNET system compared to the previous year, the performance is still not as reliable as needed.

Recently, the Crossbow EKO Wireless Sensor network has become available (Crossbow Technologies, Inc.), which operates in a true mesh-network topology, is solar powered and enables a long maintenance free operation. In addition, it has an open sensor interface to be able to integrate new types of sensors. Each EKO wireless node supports up to four sensors (soil moisture and temperature; air temperature and humidity). Sensors are plugged into the unit without the necessity to connect wires to terminal blocks or change jumper configurations. A single EKO system can support up to 35 nodes and 140 sensing points. The management of the network of EKO nodes is based on a 2.4 GHz radio/processor module. The system worked more or less flawlessly during the whole long-term experiment at CESPEVI in 2008. A reliable operating range of more than 200 m in-between two individual sensor nodes was obtained. The sensor data were made accessible on-line via the Internet, and the web-based software tool worked fine as well as the solar cell based power supply. Very infrequently a node needed to be restarted; there have been no major system failures.

Based upon these results, Balendonck and Hemming concluded (unpublished data) that this system is currently the best commercially available alternative to work with. However, in the current configuration a system like this costs about € 600 per node, including a Watermark sensor (about € 90). In the summer 2009 we plan to conduct an experiment with Crossbow EKO technology coupled to the 5TE sensor for θ, EC and T. The approximate total cost per node for this configuration is € 800 – 900, which is still too high for it to become a widespread commercial success for high density wireless sensor networks, such those required by the nurseries in Pistoia area with hundreds of irrigation plots.

Until now, the communication among RZS’, controller and irrigation valves has been assured by hydraulic or electric systems. The first one, through some pulses, and using a unidirectional communication, activated the on/off operation of the valves. Electric communication foresees the usage of wires instead of tubes and the activation of the valves takes place by the usage of solenoids. With growing field size and by having more irrigated crops, in the near future there will also be a big need to be able to control remotely valves by using a wireless system. Coates *et al.* [64] report on successful field experiments with prototypes of this equipment. Their latest technology is based upon the 900 MHz low-power wireless module (Crossbow Technologies, Inc.) [61].

Although the use of wireless systems to convey data from a RZS towards a central computer is far more widespread, recently wireless valve controllers appeared upon the market (f.i. Hunter Industries Inc.; FreeWire, Wireless Irrigation Controller, Green Wireless System, Inc.; CYCLIK, Rain Bird Corporation), and find their way, for instance into irrigation applications for agriculture and golf course management.

## 7. Conclusions

On the market there is a wide range of RZS’ that may be applied for farm level irrigation management and their selection is not simple, since several issues must been taken into consideration. A selection method has been suggested by Muňoz-Carpena [26]. It computes a final sensor value based on an array of questions regarding its attributes: measuring range, accuracy, repeatability, methodology for data communication and handling, maintenance, safety and, obviously, price.

From the viewpoint of measured quantity, two classes of RZS’ can be identified. Hydraulic tensiometers have a higher resolution in dryer media and their readings are less sensitive to the media type (no medium-specific calibration is needed). However, they require more maintenance and, in very dry soils, they fail. Low-cost dielectric tensiometer without this drawback may be available in the near future. Dielectric sensors for θ have a better resolution at higher water contents, in the θ range of 0.15 – 0.40 m^3^ m^−3^ (or even higher in artificial growing media with high porosity), and they do not need much maintenance. However, often they require a soil specific calibration and are susceptible to soil heterogeneity; therefore, they perform better in very homogeneous media like sand or media like rockwool. In general, dielectric sensors have a high repeatability (good for irrigation, which re-wets the soil), but the absolute accuracy is not very high (due to calibration errors and soil heterogeneity).

Based on the results of our experiments conducted on horticultural crops over many years, both types of RZS’ seem to perform well when appropriately integrated with irrigation controllers. However, it is undeniable that, for such a field of RZS application, accuracy is less important than repeatability, low-maintenance and easy use, and that dielectric sensors (including the dielectric tensiometer, when available) are more grower-friendly than water filled tensiometer.

Undoubtedly, the development of a number of technologies, including those involving soil moisture sensing and wireless data communication, has allowed the design and marketing of innovative irrigation controllers that may play a pivotal role in saving water and improving the economical and environmental sustainability of intensive cropping systems. In the near future, the implementation of wireless managing with information coming from GIS, interpretation of aerial photos and satellite images and topographic data will further improve the water management at farm level.

Nonetheless, in many farms, also in countries with an advanced agriculture like Italy and in high-tech production sectors such as greenhouse and nursery industry, irrigation management still depends on growers’ experience for the assessment of crop water requirements and the time of irrigation. In general, the use of RZS for irrigation scheduling in commercial cultivations is very sporadic in Italy and only very few examples exist in the sectors of grapevine, fruit crop and processing tomato cultivation. In the area of Pistoia, to our knowledge only a couple of greenhouse growers make use of RZS to control (manually, in one case) the irrigation in soil-grown ornamental crop. On the other hand, our recent and current research and demonstration activities carried out in Tuscany significantly reduced the original scepticism (especially, from the growers) on the application of RZS for efficient irrigation management of container ornamental plants. As a matter of fact, many growers are interested in such technology and are somewhat waiting for devices marketed at more affordable costs

Therefore, the main obstacles to the application of RZS technology at farm level seem to be the overall costs of such technology and the lack of effective policies for the dissemination and transfer of smart water application technology to commercial operations, including grower training. The progress in the field of electronics and computer science suggests a further reduction of prices and a more and more significant integration of advanced technologies inside the intensive agricultural sector. Moreover, ICT will assure a simplification of system functioning and an increase of the system efficiency, but with a wider use of external resources. The increase in the price of water as such and in the severity of environmental legislation concerning the exploitation and protection of water resources also will contribute to the adoption of smart water application technology.
